# Mammographic density adds accuracy to both the Tyrer-Cuzick and Gail breast cancer risk models in a prospective UK screening cohort

**DOI:** 10.1186/s13058-015-0653-5

**Published:** 2015-12-01

**Authors:** Adam R. Brentnall, Elaine F. Harkness, Susan M. Astley, Louise S. Donnelly, Paula Stavrinos, Sarah Sampson, Lynne Fox, Jamie C. Sergeant, Michelle N. Harvie, Mary Wilson, Ursula Beetles, Soujanya Gadde, Yit Lim, Anil Jain, Sara Bundred, Nicola Barr, Valerie Reece, Anthony Howell, Jack Cuzick, D. Gareth R. Evans

**Affiliations:** Centre for Cancer Prevention, Wolfson Institute of Preventive Medicine, Charterhouse Square, Barts and The London, Queen Mary University of London, London, EC1M 6BQ UK; Genesis Breast Cancer Prevention Centre and Nightingale Breast Screening Centre, University Hospital of South Manchester, Manchester, UK; Centre for Imaging Sciences, Institute for Population Health, University of Manchester, Manchester, UK; Manchester Academic Health Science Centre, University of Manchester, Manchester, UK; Arthritis Research UK Centre for Epidemiology, University of Manchester, Manchester, UK; National Institute for Health Research (NIHR) Manchester Musculoskeletal Biomedical Research Unit, Central Manchester University Hospitals NHS Foundation Trust, Manchester, UK; Institute of Cancer Sciences, University of Manchester, Manchester, UK; Education and Research Centre, University Hospital of South Manchester, Manchester, UK; The Christie NHS Foundation Trust, Manchester, UK; Institute of Human development, Genomic Medicine, Manchester Academic Health Sciences Centre, University of Manchester, Manchester, UK

## Abstract

**Introduction:**

The Predicting Risk of Cancer at Screening study in Manchester, UK, is a prospective study of breast cancer risk estimation. It was designed to assess whether mammographic density may help in refinement of breast cancer risk estimation using either the Gail model (Breast Cancer Risk Assessment Tool) or the Tyrer-Cuzick model (International Breast Intervention Study model).

**Methods:**

Mammographic density was measured at entry as a percentage visual assessment, adjusted for age and body mass index. Tyrer-Cuzick and Gail 10-year risks were based on a questionnaire completed contemporaneously. Breast cancers were identified at the entry screen or shortly thereafter. The contribution of density to risk models was assessed using odds ratios (ORs) with profile likelihood confidence intervals (CIs) and area under the receiver operating characteristic curve (AUC). The calibration of predicted ORs was estimated as a percentage [(observed vs expected (O/E)] from logistic regression.

**Results:**

The analysis included 50,628 women aged 47–73 years who were recruited between October 2009 and September 2013. Of these, 697 had breast cancer diagnosed after enrolment. Median follow-up was 3.2 years. Breast density [interquartile range odds ratio (IQR-OR) 1.48, 95 % CI 1.34–1.63, AUC 0.59] was a slightly stronger univariate risk factor than the Tyrer-Cuzick model [IQR-OR 1.36 (95 % CI 1.25–1.48), O/E 60 % (95 % CI 44–74), AUC 0.57] or the Gail model [IQR-OR 1.22 (95 % CI 1.12–1.33), O/E 46 % (95 % CI 26–65 %), AUC 0.55]. It continued to add information after allowing for Tyrer-Cuzick [IQR-OR 1.47 (95 % CI 1.33–1.62), combined AUC 0.61] or Gail [IQR-OR 1.45 (95 % CI 1.32–1.60), combined AUC 0.59].

**Conclusions:**

Breast density may be usefully combined with the Tyrer-Cuzick model or the Gail model.

**Electronic supplementary material:**

The online version of this article (doi:10.1186/s13058-015-0653-5) contains supplementary material, which is available to authorized users.

## Introduction

Breast cancer risk models estimate the chance that a woman will develop breast cancer in the future, and a more accurate assessment is needed to guide prevention and screening strategies [1]. Risk is often assessed using the Gail (or Breast Cancer Risk Assessment Tool) and Tyrer-Cuzick [or International Breast Intervention Study (IBIS)] models [2–5]. The Gail model was originally developed using a case–control study of women attending screening in the United States [4] with invasive and ductal carcinoma in situ (DCIS) cases, but the absolute rates are calibrated to invasive cancer. The Gail model is based on eight questions, including age, hormonal factors, benign disease and the number of first-degree relatives affected by breast cancer, and it has been validated to be well calibrated for the general population [6]. The Tyrer-Cuzick model was developed by pooling relative risks from overview studies and was initially used to assess eligibility for a prevention trial (IBIS-I) [5]. It is calibrated to invasive and DCIS cancer rates and includes many of the Gail risk factors, but some are handled differently, including a more complex model for family history of the disease. The Tyrer-Cuzick model has not been validated to date in a prospective screening setting, but it has been compared with the Gail model in cohorts with a strong family history [7–9].

Mammographic density appears as white (radiopaque) areas on a mammogram, and it is often measured visually as a percentage of the total breast area. Dense breasts have more fibroglandular tissue and less fat than non-dense breasts, and it is well established that women with these features are at a higher risk of breast cancer [10]. Density could be routinely measured when a woman attends screening, but it is currently not incorporated in either the Tyrer-Cuzick model or the Gail model. Some work to combine breast density with classical hormonal and familial risk factors has been based on Breast Imaging-Reporting and Data System (BI-RADS) visual density classification [11]. This has been seen to produce a relative risk of approximately 2–4-fold between the highest and lowest of four categories [12]. Results incorporating BI-RADS density into risk models have been mixed [13]. Some have concluded that BI-RADS density added minimally to the Gail model, but others have shown that it adds useful additional information to risk factors used with the Gail model [12, 14]. A limitation of BI-RADS density is that approximately 80 % women fall into the middle two categories where the risk difference is more modest [12].

Another visually assessed density measure is the percentage of the area of the breast containing fibroglandular tissue. Methods for this have been observed to produce a 4–6-fold risk difference for dense versus non-dense breasts [15], and they predict response to tamoxifen prevention [16] and both tamoxifen and aromatase inhibitors in the adjuvant setting [17, 18]. Some previous work has found continuous measures of percentage density to be useful in combination with classical risk factors. In particular, Chen et al. [19] conducted a case–control study of women recruited to a screening study in the United States during the 1970s, and Warwick et al. [20] reported a nested case–control study of women at high risk of breast cancer from the IBIS-I trial, mostly in the 1990s. Visual assessment has significant drawbacks, including the time needed if using a computer-aided system such as Cumulus [21], as well as inter- and intrareader variability [22]; however, for risk prediction, it is currently the standard by which to judge newer methods because it has consistently been shown to be a strong risk factor [15].

The objective of this study was to assess whether visually assessed percentage density might improve the Tyrer-Cuzick and Gail risk models for risk assessment of women attending screening in the United Kingdom. We did so using a prospective screening cohort of women enrolled in the Predicting Risk of Breast Cancer at Screening (PROCAS) study from Manchester, UK [23].

## Methods

### Cohort

All women invited for routine mammographic screening between October 2009 and September 2013 across 15 screening areas in Greater Manchester, UK, were mailed a questionnaire, study information and a consent form. The two-page questionnaire was designed to collect family history as well as hormonal and lifestyle risk factors for breast cancer (http://www.uhsm.nhs.uk/research/Documents/PROCAS%20Questionnaire.pdf). Each completed questionnaire was imported into a database and verified using a set of rules to check for inconsistencies. Screening mammograms were collected and stored. The earliest mammograms were film (20 %), but the majority used GE Senographe Essential full-field digital mammography (GE Healthcare, Chalfont St Giles, UK).

### Ethics, consent and permissions

Consent was obtained at the time of screening. The study was approved by Central Manchester Research Ethics Committee (reference 09/H1008/81).

### Study design

The primary clinical endpoint was diagnosis of breast cancer [International Classification of Diseases, Tenth revision, codes C50/D05: invasive breast cancer/ductal carcinoma in situ (DCIS)] from entry screen onwards, as identified through the National Health Service Breast Screening Programme (NHSBSP) system and the Somerset and North West Cancer Intelligence services. In secondary analysis, we considered invasive breast cancer only. Prospective breast cancers occurred between October 2009 and September 2014, and the median follow-up was 3.2 years. Cancers detected less than 100 days after enrolment were defined as being detected at the entry screen.

Two widely used risk models were chosen before the study began: (1) Tyrer-Cuzick (IBIS version 6.0: http://www.ems-trials.org/riskevaluator/) [4] and (2) Gail (April 2014 version: http://www.cancer.gov/bcrisktool/) [5]. They were used to assess 10-year risk. There were three limitations of the questionnaire for the models: (1) limited information regarding unaffected relatives was collected (Tyrer-Cuzick); (2) previous breast biopsy included, but not the number of biopsies (Gail); and (3) type of benign disease (including proliferative disease and atypical hyperplasia) was not recorded (Tyrer-Cuzick). Unaided visually assessed density was the primary density measure. It was assessed using all available screening mammograms (usually four: craniocaudal and mediolateral oblique images of both breast sides) and scored independently by two readers on a standard visual analogue scale from 0 % to 100 %; percentages were scanned using computer software. In total, 18 professionals assessed density; this group consisted of 10 radiologists, 2 breast physicians and 6 advanced practitioner radiographers, many of whom had participated in an earlier study of density [24].

The mean percentage from two readers and four mammogram views was used for women without breast cancer, and only the contralateral breast was used for all cancers. For practical reasons, both the left and right breasts were assessed for all participants, including those diagnosed with breast cancer at the entry screen. Mammograms were reread to assess any possible bias associated with increasing density in those thought to have cancer. Four readers who carried out the most readings reassessed the densest 101 contralateral breasts from cancers diagnosed at first screen and 101 non-cancers matched by density and year of acquisition. Each mammogram was reread by two readers independently and blinded to case status. Overall, density when reread decreased in cancers (mean 49.5–44.2 %) and non-cancers (49.8–42.4 %), which was likely due to regression to the mean from selecting the highest-density mammograms. Density dropped by 2.0 % (95 % CI −0.7 to 4.7 %) more for non-cancers than for cancers (*P* = 0.182 by Wilcoxon test), so we concluded that potential bias for risk was negligible.

### Assay methods

Tumour pathology characteristics were assessed in a standardised manner as required by pathologists reporting in the NHSBSP [25].

### Statistical analysis

The study was designed so that 600 screen-detected and interval breast cancers were expected between the first two screening rounds. This gave the study approximately 90 % power at 5 % two-sided significance for detection of an arbitrary breast cancer risk factor with a relative risk of 1.3 and occurring in 50 % of the population, or 1.5 in 30 % of the population or 2.0 in 15 % of the population.

Projected 10-year risk was taken as the primary predictor, partly because, in current UK guidelines [3], women with a 5–8 % or greater than 8 % 10-year risk would qualify for prevention and additional screening. Ten-year risk is also the default in the Tyrer-Cuzick model.

Measurement error from breast density was assessed by fitting a linear mixed-effects model by restricted maximum likelihood and corresponding intraclass correlation coefficient [26]. Breast cancer risk factors used in the models were summarised with categories for continuous factors chosen so that the reference group was an established standard or an average containing approximately the middle half of the cohort. Adjusted odds ratios (ORs) were estimated using a logistic regression with age (continuous). Percentage mammographic density was adjusted for age and body mass index (BMI) via a ‘density residual’, obtained from fitting a linear regression of density against age, BMI and type of mammogram (digital or film) [20] (see Additional file 1). This helped to make density more independent of the risk models, and combined density and risk model projections were obtained by multiplying Tyrer-Cuzick or Gail model expected risk by observed breast density risk. In analysis of risk factors, we used ORs, profile likelihood confidence intervals (CIs) and likelihood ratio (LR) χ^2^ statistics from continuous predictors [each with 1 degree of freedom (*df*)]. Logistic regression models were fitted to assess the calibration of predicted logarithmic ORs, and observed risk was the predicted risk multiplied by an estimated calibration coefficient. Observed and expected ORs were plotted using a normal kernel smoother with bandwidth chosen by 10-fold cross validation [27], also shown by decile of predicted risk. Calibration of absolute risks was not assessed. The area under the receiver operating characteristic curve (AUC) was a secondary measure of discrimination, with DeLong CIs [28]. Calibration and discrimination were also assessed by age subgroups with a likelihood-ratio test for interaction.

All *P* values were two-sided. Analysis was conducted using GNU-R version 2.15.1 statistical software [29].

## Results

### Cohort

Between October 2009 and September 2013, 201,187 women were invited to breast cancer screening and 130,332 attended (65 %). Of these, 51,744 women (40 %) consented to join the study; 750 had a breast cancer diagnosed from entry screen until the end of follow-up. To assess breast density as a risk factor, the following exclusions were made: 756 who had a previous diagnosis of breast cancer (29 with prospective cancer); 11 bilateral breast cancers and 7 for whom the side was unknown; 122 who had no visual assessment of breast density available (2 prospective cancers); 14 who were older than 73 years of age at enrolment (0 cancers); and 206 who had a breast implant (4 cancers). This left 50,628 women who were breast cancer–free before entry screen, with 697 breast cancers diagnosed after enrolment, of which 567 (81 %) were invasive, 128 were DCIS and 2 were unknown.

The majority [*n* = 556 (80 %)] of cancers were diagnosed at entry screen, 28 (4 %) between 100 days and 2.5 years after enrolment and 110 (16 %) more than 2.5 years after enrolment. For three women, the timing was unknown at the time of analysis. Ethnic or other origin was recorded for 48,807 women (96 %), of whom 46,491 (92 %) were reported as white (453 Jewish); the remainder were Asian (*n* = 739), black (*n* = 556), mixed race of ethnicity (*n* = 262) and other (*n* = 759).

The number of women per reader assessed for breast density ranged from 104 to 16,121 [interquartile range (IQR) 1600–9842]. The percentage variance in density explained by reader differences was estimated to be 11 %. Half of the absolute percentage differences between readers were less than or equal to 10.00 %; the IQR was 4.75–17.75 %. The intraclass correlation coefficient between left and right sides was 93 %.

### Analysis and presentation

Distributions of breast cancer risk factors in the cohort are shown in Table 1. In summary, the majority of the screening age cohort were postmenopausal (72 %) or perimenopausal (18 %); 8 % were currently using hormone replacement therapy. Most women were parous (87 %), on average first giving birth when aged 24 years (IQR 20–27), and most were overweight (62 % BMI >25 kg/m^2^). Twelve percent of women reported first-degree relatives with breast cancer, and 14 % disclosed a prior breast biopsy.

**Table 1:** Breast cancer risk factors in the cohort

Question	Group	No breast cancer	Breast cancer	OR (95 % CI)	Univariate summary^a^	Age-adjusted summary^a^
Age, yr	<52	10,601 (21 %)	123 (18 %)	0.85 (0.69–1.04)	58 (52–64)	
	52–64	28,466 (57 %)	387 (56 %)	1.00 (reference)	1.30 (1.14–1.48)	
	>64	10,864 (22 %)	187 (27 %)	1.27 (1.06–1.51)	15.9 (*P* < 0.001)	
Age at menarche, yr	<12	11,298 (23 %)	168 (24 %)	1.09 (0.89–1.31)	13 (12–14)	
	12–13	20,817 (42 %)	285 (41 %)	1.00 (reference)	0.99 (0.90–1.08)	0.99 (0.90–1.08)
	14+	16,685 (33 %)	230 (33 %)	1.01 (0.84–1.20)	0.1 (*P* = 0.78)	0.1 (*P* = 0.77)
	Missing	1131 (2 %)	14 (2 %)	0.90 (0.50–1.49)		
Parous	Yes	43,578 (87 %)	591 (85 %)	1.00 (reference)		
	No	6166 (12 %)	103 (15 %)	1.23 (0.99–1.51)	1.23 (0.99–1.51)	1.28 (1.03–1.58)
	Missing	187 (0 %)	3 (0 %)	1.18 (0.29–3.12)	3.6 (*P* = 0.059)	5.0 (*P* = 0.026)
Age at birth of first child, yr	<21	11,134 (26 %)	139 (24 %)	0.91 (0.74–1.11)	24 (20–27)	
	21–27	21,791 (50 %)	300 (51 %)	1.00 (reference)	1.09 (0.98–1.22)	1.13 (1.01–1.26)
	>27	10,502 (24 %)	150 (25 %)	1.04 (0.85–1.26)	2.4 (*P* = 0.12)	4.4 (*P* = 0.037)
	Missing	151 (0 %)	2 (0 %)	0.96 (0.16–3.03)		
Age at menopause, yr	<46	16,201 (47 %)	242 (47 %)	0.73 (0.56–0.93)	50 (45–51)	
	46–52	7933 (23 %)	86 (17 %)	1.00 (reference)	1.30 (1.16–1.46)	1.28 (1.14–1.44)
	>52	4906 (14 %)	90 (18 %)	1.23 (0.96–1.56)	21.4 (*P* < 0.001)	18.4 (*P* < 0.001)
	Missing	5321 (15 %)	95 (19 %)	1.20 (0.94–1.51)		
BMI, kg/m^2^	<25	17,538 (35 %)	216 (31 %)	1.00 (reference)	26.5 (23.7–30.4)	
	25–30	16,633 (33 %)	241 (35 %)	1.18 (0.98–1.42)	1.10 (1.01–1.21)	1.11 (1.01–1.21)
	30+	12,442 (25 %)	192 (28 %)	1.25 (1.03–1.52)	4.6 (*P* = 0.033)	4.8 (*P* = 0.028)
	Missing	3318 (7 %)	48 (7 %)	1.17 (0.85–1.59)		
BMI premenopause, kg/m^2^	<25	1710 (38 %)	18 (31 %)	1.00 (reference)	26.2 (23.3–30.1)	
	25–30	1394 (31 %)	24 (41 %)	1.64 (0.89–3.07)	0.98 (0.71–1.33)	0.98 (0.70–1.32)
	30+	1087 (24 %)	14 (24 %)	1.22 (0.60–2.46)	0.0 (*P* = 0.92)	0.0 (*P* = 0.88)
	Missing	322 (7 %)	3 (5 %)	0.89 (0.21–2.63)		
BMI perimenopause, kg/m^2^	<25	3176 (36 %)	33 (34 %)	1.00 (reference)	26.4 (23.5–30.3)	
	25–30	2816 (32 %)	33 (34 %)	1.13 (0.69–1.84)	1.00 (0.77–1.27)	1.00 (0.77–1.27)
	30+	2151 (25 %)	25 (26 %)	1.12 (0.66–1.88)	0.0 (*P* = 0.99)	0.0 (*P* = 0.99)
	Missing	601 (7 %)	7 (7 %)	1.12 (0.45–2.40)		
BMI postmenopause, kg/m^2^	<25	11,918 (35 %)	157 (31 %)	1.00 (reference)	26.5 (23.8–30.4)	
	25–30	11,767 (34 %)	176 (34 %)	1.14 (0.91–1.41)	1.13 (1.02–1.25)	1.14 (1.02–1.26)
	30+	8603 (25 %)	144 (28 %)	1.27 (1.01–1.60)	5.2 (*P* =0.023)	5.5 (*P* = 0.019)
	Missing	2073 (6 %)	36 (7 %)	1.32 (0.90–1.88)		
First-degree relatives with breast cancer	0	44,269 (89 %)	595 (85 %)	1.00 (reference)		
	1	5257 (11 %)	92 (13 %)	1.30 (1.04–1.62)	1.32 (1.09–1.58)	1.30 (1.07–1.56)
	2+	405 (1 %)	10 (1 %)	1.84 (0.91–3.27)	7.8 (*P* =0.005)	7.0 (*P* = 0.008)
Biopsies	No	41,311 (83 %)	530 (76 %)	1.00 (reference)		
	Yes	7174 (14 %)	136 (20 %)	1.48 (1.22–1.78)	1.48 (1.22–1.78)	1.45 (1.19–1.75)
	Missing	14,46 (3 %)	31 (4 %)	1.67 (1.14–2.37)	15.0 (*P* < 0.001)	13.6 (*P* <0.001)
Current HRT by age group, yr, yes/no	<58, no	23,758 (48 %)	286 (41 %)	1.00 (reference)		
	<58, yes	2529 (5 %)	29 (4 %)	0.95 (0.64–1.37)	1.12 (0.82–1.49)	1.43 (1.03–1.95)
	58+, no	22,328 (45 %)	354 (51 %)	1.32 (1.13–1.54)	0.5 (*P* = 0.47)	4.7 (*P* = 0.031)
	58+, yes	1316 (3 %)	28 (4 %)	1.77 (1.17–2.57)		

*HRT* hormone replacement therapy, *BMI* body mass index, *OR* odds ratio, *CI* confidence interval

^a^Summary, first row: median [interquartile rage (IQR)], second row: IQR odds ratio (95 % confidence interval), third row: likelihood ratio χ^2^ (*P*-value)

The risk models provided useful information for discrimination (Table 2). The IQR-ORs were 1.22 for the Gail model and 1.36 for Tyrer-Cuzick, but the AUCs were modest at 0.55 and 0.57, respectively. The Tyrer-Cuzick model had more than twice the amount of information as the Gail model in terms of likelihood ratio χ^2^ (49.2 vs 19.7, respectively). However, the Gail model performed better for cancers detected after entry screen (see Additional file 1), for which the IQR-OR was 1.35 (95 % CI 1.11–1.62) compared with 1.36 (95 % CI 1.12–1.63) for the Tyrer-Cuzick model. The findings were not materially affected when we restricted attention to invasive cancer (Table 2).

**Table 2:** Performance of risk models and breast density

	No cancer, median (IQR)	Cancer, median (IQR)	IQR-OR^a^ (95 % CI)	IQR-OR^b^ (95 % CI)	LR^a^-χ^2^	LR^b^-Δχ^2^	AUC^a^ (95 % CI)	AUC^b^ (95 % CI)
Primary: invasive + DCIS								
Number of women	49,931	697						
Gail	3.50 % (2.90–4.40 %)	3.70 % (3.10–4.60 %)	1.22 (1.12–1.33)	1.21 (1.10–1.31)	19.7		0.55 (0.52–0.57)	
Density residual	−0.06 (−0.73–0.63)	0.24 (−0.40–0.91)	1.48 (1.34–1.63)	1.47 (1.33–1.62)	61.4	58.6	0.59 (0.57–0.61)	0.59 (0.57–0.61)
Tyrer-Cuzick	2.66 % (2.12–3.47 %)	2.94 % (2.28–3.97 %)	1.36 (1.25–1.48)	1.34 (1.23–1.45)	49.2		0.57 (0.55–0.59)	
Density residual	−0.06 (−0.73–0.63)	0.24 (−0.40–0.91)	1.48 (1.34–1.63)	1.45 (1.32–1.60)	61.4	54.8	0.59 (0.57–0.61)	0.61 (0.59–0.63)
Secondary: invasive								
Number of women	50,061	567						
Gail	3.50 % (2.90–4.40 %)	3.70 % (3.00–4.55 %)	1.19 (1.07–1.31)	1.17 (1.06–1.29)	11.3		0.54 (0.52–0.56)	
Density residual	−0.06 (−0.73–0.63)	0.24 (−0.40–0.85)	1.47 (1.32–1.64)	1.46 (1.31–1.63)	48.5	46.6	0.59 (0.56–0.61)	0.59 (0.57–0.61)
Tyrer-Cuzick	2.66 % (2.12–3.47 %)	2.93 % (2.29–3.88 %)	1.33 (1.21–1.46)	1.30 (1.18–1.43)	33.6	33.6	0.57 (0.55–0.59)	
Density residual	−0.06 (−0.73–0.63)	0.24 (−0.40–0.85)	1.47 (1.32–1.64)	1.46 (1.31–1.63)	48.5	43.7	0.59 (0.56–0.61)	0.61 (0.58–0.63)

*Gail* 10-year risk, *TC* Tyrer-Cuzick 10-year risk, *DR* density residual, *IQR* interquartile range, *OR* odds ratio, *CI* confidence interval, *LR* likelihood ratio, *AUC* area under the receiver operating characteristic curve

^a^Univariate

^b^Multivariate (risk model + density)

**Fig. 1:**
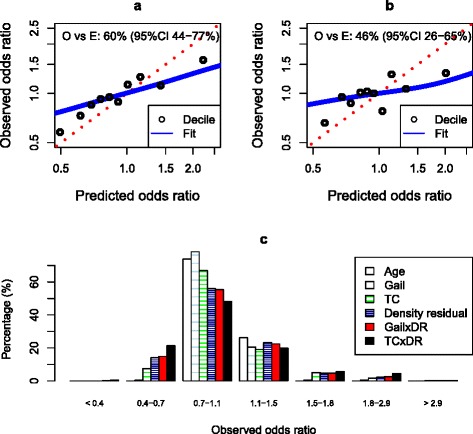
Calibration and spread of risk from the models and density. The predicted and observed odds ratios from (**a**) the Tyrer-Cuzick model and (**b**) the Gail model in the cohort are shown. **c** Histogram of observed risk. O vs E is the estimate from a logistic regression of the logarithmic predicted odds ratio. *TC* Tyrer-Cuzick 10-year risk, *DR* density residual

ORs derived from 10-year risks were not well calibrated, being 60 % (95 % CI 44–74 %) of expected for Tyrer-Cuzick and 46 % (95 % CI 26–65 %) of expected for the Gail model. Figure 1a, b provides a graphical illustration in which the shrinkage in risk distribution is reflected by the line of fit and risk deciles are less than expected for the high-risk groups and more than expected for the low-risk groups.

Overall, the Gail model was not more informative than age (AUC 0.55, 95 % CI 0.52–0.57). However, Table 3 shows that it added independent information to age (LR χ^2^ 9.2), similarly for Tyrer-Cuzick (LR χ^2^ 48.8). There was little evidence of interaction by age group [Gail LR χ^2^ 3.9 (*df* = 5), *P* = 0.57; Tyrer-Cuzick, LR χ^2^ 5.8 (*df* = 5), *P* = 0.33].

**Table 3:** Model performance, by age group

		Calibration: O/E (95 % CI)		Discrimination: AUC (95 % CI)	
Age group, yr	Cases/total (%)	Tyrer-Cuzick	Gail	Tyrer-Cuzick	Gail
47–49	170/13,662 (1.2 %)	51 % (16–85 %)	43 % (−4–88 %)	0.57 (0.52–0.61)	0.54 (0.50–0.59)
50–54	125/10,677 (1.2 %)	52 % (14–89 %)	44 % (−8–93 %)	0.55 (0.49–0.60)	0.54 (0.49–0.59)
55–59	42/4312 (1.0 %)	70 % (−2–138 %)	33 % (−75–135 %)	0.58 (0.49–0.67)	0.53 (0.45–0.62)
60–64	147/8244 (1.8 %)	79 % (42–114 %)	17 % (−33–64 %)	0.62 (0.58–0.66)	0.52 (0.48–0.57)
65–69	173/10,926 (1.6 %)	44 % (10–76 %)	17 % (−29–60 %)	0.54 (0.50–0.59)	0.51 (0.46–0.55)
70–73	40/2807 (1.4 %)	122 % (54–188 %)	99 % (19–172 %)	0.64 (0.55–0.73)	0.58 (0.48–0.68)

*O/E* observed to expected odds ratio, *CI* confidence interval, *AUC* area under the receiver operating characteristic curve

Visually assessed breast density was inversely correlated with BMI (Spearman correlation coefficient −0.38) and age (Spearman correlation coefficient −0.19). Density was less for digital mammograms (median 24 %, IQR 14–37 %) than for film (27 %, 15–44 %) (*P* < 0.001). It was higher in women with cancer (median 28 %, IQR 18–41 %) than in those without (24 %, 14–38 %) (*P* < 0.001). After adjusting density for age, BMI and type of mammogram in the density residual, we observed that the LR χ^2^ was doubled from 27.4 to 61.4, and it was a stronger univariate risk factor than either model (IQR-OR 1.48, AUC 0.59).

Mammographic density added substantial significant information to the models and increased the AUC by 0.04 for both (Table 2). An OR relative to the sample mean was estimated for each woman from the risk models alone and in combination with breast density. Figure 1c shows the distributions of ORs, which demonstrate that more women were accurately given high and low risks when density was added. Table 4 cross-tabulates incidence by risk groups from the models alone and with density. Inspection similarly shows that adding density helped to identify more high- and low-risk women accurately and that the number of women with a predicted Tyrer-Cuzick 10-year risk greater than 8 % was more than doubled, from 1.2 % to 2.7 %.

**Table 4:** Breast cancer incidence cross-classified by 10-year risk groups from the Tyrer-Cuzick and Gail models when combined with breast density

	Risk model combined with density (10-year risk)						
Risk model	<1 %	1–2 %	2–3.5 %	3.5–5 %	5–8 %	>8 %	Total
Tyrer-Cuzick							
<1 %	0/60 (0.0 %)	**0/18 (0.0 %)**					0/78 (0.0 %)
1–2 %	4/700 (0.6 %)	60/6910 (0.9 %)	**18/1976 (0.9 %)**	**2/85 (2.4 %)**	**0/8 (0.0 %)**		84/9679 (0.9 %)
2–3.5 %	0/6 (0.0 %)	69/7425 (0.9 %)	221/16,515 (1.3 %)	**75/3807 (2.0 %)**	**16/652 (2.5 %)**	**1/24 (4.2 %)**	382/28,429 (1.3 %)
3.5–5 %		0/29 (0.0 %)	21/2689 (0.8 %)	66/3139 (2.1 %)	**35/1508 (2.3 %)**	**2/139 (1.4 %)**	124/7504 (1.7 %)
5–8 %			3/144 (2.1 %)	20/1181 (1.7 %)	48/2257 (2.1 %)	**16/758 (2.1 %)**	87/4340 (2.0 %)
>8 %				0/2 (0.0 %)	5/172 (2.9 %)	15/424 (3.5 %)	20/598 (3.3 %)
Total	4/766 (0.5 %)	129/14,382 (0.9 %)	263/21324 (1.2 %)	163/8214 (2.0 %)	104/4597 (2.3 %)	34/1345 (2.5 %)	697/50,628 (1.4 %)
Gail							
<1 %							
1–2 %	0/17 (0.0 %)	5/601 (0.8 %)	**5/257 (1.9 %)**	**0/14 (0.0 %)**			10/889 (1.1 %)
2–3.5 %	0/1 (0.0 %)	33/4286 (0.8 %)	170/14,115 (1.2 %)	**62/3894 (1.6 %)**	**15/756 (2.0 %)**	**0/42 (0.0 %)**	280/23,094 (1.2 %)
3.5–5 %		1/90 (1.1 %)	68/7048 (1.0 %)	118/7751 (1.5 %)	**75/3533 (2.1 %)**	**11/315 (3.5 %)**	273/18,737 (1.5 %)
5–8 %			3/277 (1.1 %)	21/1851 (1.1 %)	63/3610 (1.7 %)	**20/1105 (1.8 %)**	107/6843 (1.6 %)
>8 %				0/15 (0.0 %)	8/316 (2.5 %)	19/734 (2.6 %)	27/1065 (2.5 %)
Total	0/18 (0.0 %)	39/4977 (0.8 %)	246/21,697 (1.1 %)	201/13,525 (1.5 %)	161/8215 (2.0 %)	50/2196 (2.3 %)	697/50,628 (1.4 %)

Bold cells indicate combinations that increased risk when density was added

**Fig. 2:**
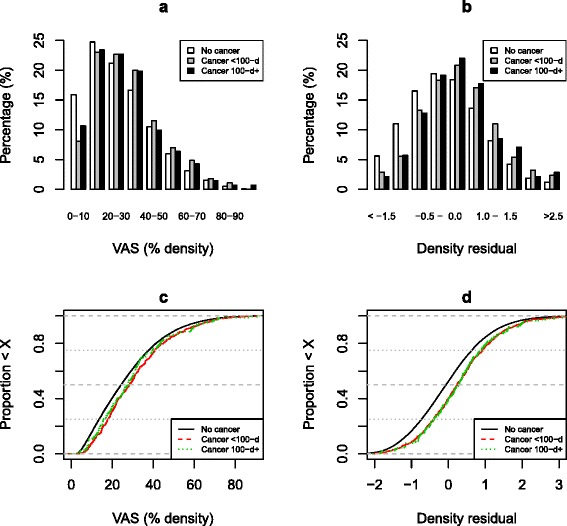
Breast density and residual by time of diagnosis since enrolment. **a** and **c** Histograms and empirical cumulative distribution functions for breast density. **b** and **d** Histograms and empirical cumulative distribution functions for age, body mass index and type of image adjusted residual. The cancers are split into those diagnosed within 100 days of entry (<100-d) and more than 100 days (100-d+). A Wilcoxon test for the difference between <100 days and 100+ days yielded *P* = 0.34 for visual analogue scale (VAS) and *P* = 0.98 for the residual.

Most (80 %) of the cancers were diagnosed at entry, but the distribution of breast density and residual was similar in cancers diagnosed at screening versus later (Fig. 2). Furthermore, the IQR-ORs for adjusted density were 1.48 (95 % CI 1.32–1.64) at entry screen and 1.49 (95 % CI 1.20–1.85) thereafter (see Additional file 1). This suggests that density was predictive of future cancer, as well as being a cross-sectional risk factor.

## Discussion

The results of univariate analysis of breast cancer risk factors shown in Table 1 broadly agreed with the literature [5]. In this predominately postmenopausal cohort, age at menopause yielded the most information on the basis of LR χ^2^ statistics. Age at menopause is not in the Gail model, so this is part of the reason why overall the Tyrer-Cuzick model performed better. It was also interesting to note that a self-reported previous biopsy conferred more information than did presence of affected first-degree relatives. Mammographic density was a stronger risk factor than the risk factors used by the Tyrer-Cuzick or Gail models, after adjustment for age and BMI.

Although the risk models were found to provide useful information for risk stratification without mammographic density, their relative risks were not well calibrated. A partial reason is that some risk factors were not as strong as expected, the most noticeable being family history [30].

Our study differs from some other work in that we used a percentage visual assessment rather than the four-category BI-RADS scale. An advantage is that the visual percentage is not designed to assess masking issues, where dense tissue may make it more difficult to detect cancer in mammograms. This is relevant because in the most recent version (fifth edition) of the BI-RADS scale, specific reference to percentage density was removed [11] to focus on masking issues. It remains to be seen if the change in emphasis and focus on masking affect the distribution of BI-RADS density, as well as its relationship with risk.

Earlier studies have also shown that continuous measures of percentage density are useful in combination with classical risk factors. Chen et al. [19] used a case–control study of women (majority aged 35–74 years) recruited into a screening study in the United States during the 1970s and proposed a better model with some of the Gail model factors, weight and categorised percentage density. While their study demonstrated that density is very useful after allowing for classical factors, the model does not appear to be widely adopted in clinical practice, nor does it seem to have been externally validated. Warwick et al. [20] showed that density added to the Tyrer-Cuzick model in a case–control analysis of relatively young women (90 % aged 40–60 years), mostly from the United Kingdom, who were at high risk of breast cancer, which is a different source population than targeted in our present study. An issue that affects the earlier studies is that they recruited when film rather than digital mammography was used, and when populations were less obese. Our work adds to this literature by measuring how well commonly used risk estimation models performed in a UK screening cohort, and by showing how these models might be improved with breast density. The findings might help to inform the design of risk-adapted screening and prevention strategies in the United Kingdom and elsewhere.

Some limitations of the study include the following. Firstly, the risk factors were self-reported via a questionnaire. Secondly, breast density was used to predict risk at the same screen, and the subgroup analysis by time of diagnosis since enrolment was limited. More follow-up is needed to help to address this issue. Thirdly, the visually assessed score required human judgement, which might make it unreliable for routine use in a screening program [31], although the same applies to BI-RADS density. Exploration of automated methods is ongoing in a subset of the cohort. Fourthly, a possible limitation is that the reader was able to partially identify which mammograms had cancer at baseline. However, a small study suggested such bias is negligible, and almost identical relationships with breast cancer risk were observed in the cancers diagnosed after the entry screen, which was also found in an earlier screening study of the same density measure [32]. Thus, we believe this bias is extremely small, if present at all.

Two further criticisms might be made of the study. Firstly, it might be argued that the Gail model predicts the absolute risk of invasive cancer and does not include DCIS. However, the relative risk model was fitted to invasive cancer and DCIS [4], which is partial justification for using this endpoint in the analysis. It was also specified before inspection of the data in a statistical analysis plan that focused on relative risks rather than absolute rates, and a secondary analysis of invasive cancers did not suggest a bias against the Gail model from including DCIS. Secondly, one might argue that the risk models were designed to predict only future risk of women with a negative mammogram. We feel that it is not unreasonable to apply the implied relative risks from risk models to a cross-sectional study, albeit with a recalibration, because previous work has found breast cancer risk factors to have a similar magnitudes in case–control studies as for cohorts (see, e.g., [30]). Further, many of the participants will have had a previous negative screening mammogram, and we did not find a significant interaction between model performance and age.

## Conclusions

We used a prospective cohort from the United Kingdom to test whether visually assessed mammographic density added to the Tyrer-Cuzick and Gail models for women who attend breast cancer screening. Discrimination measured by changes in LR χ^2^ was doubled, and a larger proportion of women could be accurately classified to be at more than a moderately high risk when density was combined with either model. However, the AUC values remained modest, so there is still more to be done.

To our knowledge, this study is the first in which the Tyrer-Cuzick risk model has been evaluated in a prospective screening setting. Although the analysis was limited by the cross-sectional nature and issues such as lead time, the Tyrer-Cuzick model was found to provide useful information for risk assessment. The Gail model has been well validated for use in North America, but in this UK setting the Gail model was outperformed by the Tyrer-Cuzick model.

In conclusion, the data in this report are of relevance for designing improvements to the national screening program in the United Kingdom. By combining the Tyrer-Cuzick model with breast density, we identified 72 % of the population which had an average or below average risk of breast cancer, and they had proportionally fewer breast cancers. Such results might be used to inform modelling of the effect of different strategies for risk-adapted screening and prevention.

## Additional file

Additional file 1: Appendix 1. Density residual: description of methods. **Appendix 2: Table S1.** Univariate and multivariate performance of breast density and the Tyrer-Cuzick and Gail risk models, subgroup analysis by time of cancer diagnosis. (PDF 396 kb)

## Abbreviations

AUC: area under the receiver operating characteristic curve; Gail model
BI-RADS: Breast Imaging-Reporting and Data System
BMI: body mass index
CI: confidence interval
DCIS: ductal carcinoma in situ
*df*: degrees of freedom
DR: density residual
Gail: 10-year risk
HRT: hormone replacement therapy
IBIS: International Breast Intervention Study
IQR: interquartile range
IQR-OR: interquartile range odds ratio
LR: likelihood ratio
NHSBSP: National Health Service Breast Screening Programme
O/E: observed vs expected
OR: odds ratio
PROCAS: Predicting Risk of Breast Cancer at Screening study
TC: Tyrer-Cuzick 10-year risk
VAS: visual analogue scale
