# Supplementary material for: Mammographic density adds accuracy to both the Tyrer-Cuzick and Gail breast cancer risk models in a prospective UK screening cohort
Source: Breast Cancer Res. 2015 Dec 1;17:147. doi: 10.1186/s13058-015-0653-5 (PMC4665886; doi:10.1186/s13058-015-0653-5)
Supplement: Additional file 1: Appendix 1. — Density residual: description of methods. Appendix 2: Table S1. Univariate and multivariate performance of breast density and the Tyrer-Cuzick and Gail risk models, subgroup analysis by time of cancer diagnosis. (PDF 396 kb) [file 13058_2015_653_MOESM1_ESM.pdf]

Additional material

Appendix

Density residual

The approach to adjust for age and BMI formed a density residual r_i for each woman $i=1,\ldots,n$ of the form

$$r_i = z_i - (a + b_1 x_{i1} + b_2 x_{i2} + b_3 x_{i3})$$

where $a, b_1, b_2,$ and b_3 are regression parameters; x_{i1} = age minus 47, x_{i2} = BMI minus 27.5 (mean BMI), and x_{i3}=1 if a digital mammogram was used and 0 otherwise. Percent density d_i was included through the transformation from Warwick *et al.* [25] of $t_i = log\{ d_i^* / (1-d_i^*) \}$ where $d_i^* = (0.025+0.95\ d_i)^{0.5}$ and $z_i = t_i$ / sd(t) was normalised to have unit variance by dividing by the observed standard deviation (sd). The density residual was obtained by fitting the linear regression model to the cohort by ordinary least squares. Age and mammogram type were available for all women, and missing BMI was dropped from estimation; mean BMI (27.5) was imputed for these women to obtain their residual. The point estimate of the intercept a = 0.263, age parameter b_1= -0.019, BMI parameter b_2 = -0.046 and type of mammogram parameter b_3 = 0.119.

Supplementary Table

Table S1: Univariate and multivariate performance of breast density and the Tyrer-Cuzick and Gail risk models, sub-group analysis by time of cancer diagnosis. Three cancers where date of diagnosis was not available are excluded. The odds ratio is for the difference between the upper and lower quartiles in the cohort.

Factor	N (no BC)	(BC)	OR (95% CI)	LR-χ^2	AUC (95% CI)	P
(i) < 100 days						
(a) Univariate						
Gail 10-yr	49931	556	1.20 (1.09 - 1.32)	12.7	0.54 (0.51-0.56)	3.72e-04
T-C 10-yr	49931	556	1.37 (1.25 - 1.51)	41.5	0.57 (0.55-0.60)	1.17e-10
Density (%)	49931	556	1.33 (1.19 - 1.48)	24.6	0.57 (0.55-0.59)	7.09e-07
- digital	41496	434	1.34 (1.18 - 1.51)	19.8	0.57 (0.54-0.59)	8.39e-06
- other	8435	122	1.24 (0.96 - 1.58)	2.8	0.55 (0.50-0.60)	9.6e-02
DR	49931	556	1.48 (1.32 - 1.64)	48.2	0.59 (0.56-0.61)	3.78e-12
(b) Multivariate						
Gail 10-yr	49931	556	1.18 (1.07 - 1.30)	12.7	0.55 (0.52-0.57)	3.72e-04
DR with Gail			1.47 (1.31 - 1.63)	46.2	0.59 (0.57-0.61)	1.06e-11
T-C 10-yr	49931	556	1.35 (1.22 - 1.48)	41.5	0.57 (0.55-0.59)	1.17e-10
DR with T-C			1.45 (1.30 - 1.61)	42.8	0.61 (0.59-0.63)	5.95e-11
(ii) 100 days+						
(a) Univariate						
Gail 10-yr	49931	138	1.35 (1.11 - 1.62)	9.0	0.58 (0.53-0.62)	2.63e-03
T-C 10-yr	49931	138	1.36 (1.12 - 1.63)	9.5	0.58 (0.54-0.63)	2.1e-03
Density (%)	49931	138	1.22 (0.97 - 1.52)	2.9	0.54 (0.50-0.59)	8.79e-02
- digital	41496	116	1.27 (0.99 - 1.61)	3.6	0.55 (0.50-0.60)	5.92e-02
- other	8435	22	1.05 (0.56 - 1.86)	0.0	0.51 (0.38-0.63)	8.75e-01
DR	49931	138	1.49 (1.20 - 1.85)	12.8	0.59 (0.54-0.63)	3.39e-04
(b) Multivariate						
Gail 10-yr	49931	138	1.33 (1.10 - 1.60)	9.0	0.55 (0.52-0.57)	2.63e-03
DR with Gail			1.48 (1.19 - 1.83)	12.0	0.59 (0.57-0.61)	5.41e-04
T-C 10-yr	49931	138	1.33 (1.09 - 1.60)	9.5	0.57 (0.55-0.59)	2.1e-03
DR with T-C			1.46 (1.18 - 1.81)	11.5	0.61 (0.59-0.63)	6.91e-04

T-C: Tyrer-Cuzick; DR: Density residual
